# The effect of various extraction techniques on the quality of sage (*Salvia officinalis* L.) essential oil, expressed by chemical composition, thermal properties and biological activity

**DOI:** 10.1016/j.fochx.2022.100213

**Published:** 2022-01-19

**Authors:** Saša Đurović, Darko Micić, Lato Pezo, Danka Radić, Julia G. Bazarnova, Yulia A. Smyatskaya, Stevan Blagojević

**Affiliations:** a Institute of General and Physical Chemistry, Studentski trg 12, 11158 Belgrade, Serbia; b Peter the Great Saint-Petersburg Polytechnic University, Graduate School of Biotechnology and Food Industries, Polytechnicheskaya street, 29, 195251 Saint-Petersburg, Russia

**Keywords:** Sage, Extraction conditions, Extraction techniques, Essential oil quality, Chemical composition, Thermal properties, Biological activity, Artificial neural network

## Abstract

In this study, influence of the extraction techniques on the quality of the sage essential oil was investigated. Obtained samples were analyzed for chemical composition by GC/MS, thermal properties by thermogravimetric analysis (TGA), and for biological activity: antioxidant (DPPH, CUPRAC, FRAP, ABTS, HRSA and TBARS), microbiological (*Staphylococcus aureus*, *Escherichia coli*, *Bacillus subtilis*, *Pseudomonas aeruginosa, Candida albicans*, and *Aspergillus niger*), and cytotoxic (HeLa, LS-174, A549 and MRC-5) activities. Chemical composition showed that viridiflorol was principal compound in all samples followed by camphor, thujones, and verticiol. MWD 400 W was the most potent antioxidant agent, D 200 W and MWD 400 W antimicrobial agents, while hydrodistallates (D 200 W and D 400 W) were the most potent cytotoxic agents. An artificial neural network model was developed for the antioxidant activity anticipation of analyzed samples. These models showed good prediction properties (the *r^2^* value during training cycle for output variables was 0.998).

## Highlights

• Sage essential oils was isolated by different extraction techniques.
• Principal compound was viridiflorol followed by camphor, thujenes, and verticiol.
• Samples were assessed for antioxidant, antimicrobial, and cytotoxic activities.
• Obtained essential oils were investigated for thermal behavior by TGA analysis.
• ANN model was developed, for the anticipation of antioxidant activity.

## 1 Introduction

Medicinal herbs are known and used for the treatment of various diseases and disorders since ancient times. Such extensive application of these plants is because of the presence of the various chemical compounds which are beneficial for human health. Awareness about the existence of such compounds in the plants dramatically increased the popularity of tea as a drink all around the globe (Vidović et al., 2013). The beverage itself is preparing using almost every part of the plant including fruits, leaves, flowers, and sometimes seeds and stems, while preparation is quite simple and includes pouring the hot water over the plant material allowing the steeping process for 5–10 min (Chan et al., 2010, Piljac-Žegarac et al., 2013). It is worth mentioning that preparation relies on medical purposes as well as on tradition. Thus, tea may be made by multiple infusions, using water heated at different temperatures, or with milk, honey, lemon, and/or sugar (Belščak et al., 2011). After steeping for a certain period in hot water, biologically active compounds such as terpenes and polyphenols dissolved in the water. Their presence is very significant because they are very potent antioxidant agents which are able to neutralize reactive oxygen (ROS) (Aoshima et al., 2007, Atoui et al., 2005). Besides the above-mentioned antioxidant activity, compounds in plants possess a wide range of another biological potency such as antimicrobial, anti-carcinogenic, antiviral, anti-inflammatory, anti-allergic, immune-stimulatory, and estrogenic activities (Čestic et al., 2016).

Sage (*Salvia officinalis* L.) is one of about 900 species from the genus *Salvia*. It is a perennial, evergreen shrub native to Mediterranean and Middle East areas (Radivojac et al., 2020, Vidović et al., 2013). This plant is well known as a medicinal plant, used for the treatment of several diseases and disorders, such as depression, obesity, diabetes, lupus, dementia, cancer, and heart diseases (Hamidpour et al., 2014). According to the many research, sage has antimicrobial action against various Gram-positive (*Streptococcus* sp. *Bacillus* sp., etc.) and Gram-negative (*Escherichia coli*, *Klebsiella pneumonia, Pseudomonas* sp., etc*.*) bacteria, and fungi (Císarová et al., 2018).

Besides the application in medicine, this plant is used in phytopharmacy, the cosmetic industry, and food flavoring (Bozin et al., 2007). Sage is also known as an aromatic plant, which means it contains a significant amount of essential oil (0.7–5.2%). Essential oils, which mainly contain volatile organic compounds known as terpenes, are also known and widely investigated because of their biological activity such as antioxidant, antimicrobial, anti-virulent, anti-parasitical, insecticidal, etc. (Radivojac et al., 2020). Previous studies identified about 120 different compounds in sage essential oil, while the principal compounds were monoterpenes thujenes (both α- and β-thujenes), borneol, eucalyptol (1,8-cineole), and borneol, with the occurrence of the sesquiterpenes α-caryophyllene (humulene) and β-caryophyllene (Ghorbani & Esmaeilizadeh, 2017).

The objective of this study is to investigate the influence of the extraction techniques on the chemical profile, thermal behaviour, and biological activity of sage essential oil. For such purposes, two extraction techniques (hydrodistillation and microwave-assisted hydrodistillation) were used for the isolation of essential oils. Obtained samples were tested for antioxidant, antimicrobial, and cytotoxic activities by different *in vitro* assays. Furthermore, another objective of this study was to investigate the potential to anticipate the antioxidant activity of sage essential oils, depending on the content of bioactive compounds obtained in samples. This investigation was investigated according to Yoon's interpretation method (Yoon et al., 1993), performed using the developed artificial neural network model.

## 2 Material and methods

### 2.1 Plant material

For this study, leaves of the sage (*Salvia officinalis* L.) was selected for isolation of the essential oil. Plant material was acquired from the Institute for Medical Plant Research “Dr. Josif Pancic” (Belgrade, Republic of Serbia) in 2020. Plant material was kept in the paper bags in shade and at a constant temperature. The leaves were grounded in the blender prior to the essential oil isolation. The mean particle size (0.6034 mm) was determined by sieve set (CISA Cedaceria Industrial, Barcelona, Spain).

### 2.2 Isolation of the essential oil

Isolation of the essential oil (EO) from the sage plant material was done by using previously described classical hydrodistillation and microwave-assisted hydrodistillation (Radivojac et al., 2020). Hydrodistillation was done at 200 and 400 W (D 200 W and D 400 W), while microwave-assisted hydrodistillation was performed at 200 (MWD 200 W), 400 (MWD 400 W), 600 (MWD 600 W), and 800 W (MWD 800 W).

### 2.3 Chemical profile

Chemical profile of EO samples was done by using Thermo Fisher Focus GC coupled with Polaris Q mass spectrometer according to the previously reported (Šojić et al., 2019) and fully described method (Micić et al., 2021). Dissolved samples were injected into the GC through TriPlus autosampler (2 µL) into the TR WAX-MS column (30 m × 0.25 mm, 0.25 µm). Compounds were identified by comparing their mass spectra with mass spectra in NIST 2011 database and MS spectra obtained after injecting the standards. Results were expressed as relative percentage (%) and as milligram of analyzed compound per gram of EO (mg/g).

### 2.4 Thermal analysis

TA Instruments TGA Q500 Thermogravimetric Analyzer (Delaware, USA) was used for the thermal analysis of sage EOs. Samples (10.0 ± 0.5 mg) were heated from ambient temperature to 200 °C, with heating rate of 5 °C/min, under nitrogen purge flow of 60 mL/min. TA Advantage Universal analysis 2000 software was used to process all thermograms.

### 2.5 Biological activity

#### 2.5.1 Antioxidant activity

Antioxidant activity of the obtained EOs was determined by using 5 different *in vitro* assays (DPPH, CUPRAC, FRAP, ABTS, HRSA, and TBARS). The DPPH assay is based on the scavenging of stable DPPH radical and was conducted according to the previously described method (Espín et al., 2000) with slight modification and adaptation for analysis of the EOs (Zeković et al., 2016). CUPRAC (cupric ion reducing antioxidant capacity) relies on the reduction of Cu (II) ions to Cu (I) ion which is followed by the color change into orange-yellow. Measurements were done according to the previously described method (Özyürek et al., 2011). FRAP (ferric antioxidant power reduction) is essentially the electron transfer process (Cerretani & Bendini, 2010). The ABTS test is the reaction of the conversion of the ABTS^+^ into neutral ABTS which is followed by discoloration (Zhen et al., 2016). HRSA (hydroxy radical scavenging assay) is the scavenging of the hydroxy radicals formed during the Fenton reaction. The test was conducted according to the previously described method (Sundararajan et al., 2018). TBARS (thiobarbituric acid reactive substances) assay measures the amount of malonaldehyde presented in the sample and generated during the lipid peroxidation. Assay was conducted according to the previously reported method (Falowo et al., 2019). All results were expresses as IC_50_ values (µg/mL) indicating the amount of the sample required to neutralize 50% of radical species.

#### 2.5.2 Antimicrobial activity

Antimicrobial activity was determined by disc diffusion method against 7 microbial strains: *Candida albicans* (ATCC 10231), *Staphylococcus aureus* (ATCC 25923), *Escherichia coli* (ATCC 25922), *Bacillus subtilis* (ATCC 6633), *Pseudomonas aeruginosa* (ATCC 27853), and *Aspergillus niger* (ATCC 16404). Assessment of the antimicrobial activity was done according to the previously described procedure (Pavlić et al., 2017).

#### 2.5.3 Cytotoxic activity

Cytotoxic activity was assessed according to the previously reported MTT assay (Radojković et al., 2016). Samples were tested against 4 cell lines: cells derived from cervical cancer cells (HeLa), cells derived from human colon cancer (LS-174), adenocarcinomic human alveolar basal epithelial cells (A549), and normal cell line (MRC-5). Results are expressed as IC_50_ values in µg/mL.

### 2.6 Statistical analysis

All experiments were run in triplicate. The presented results are formatted as: means ± standard deviation (±SD, n = 3). The post – hoc Tukey’s HSD test was implemented to test the differences between means of samples. The estimations were done using Statistica 2010 software. Principal Component Analysis (PCA) was used for evaluation of the influence of bioactive compounds content, antioxidant activity, microbiological data and cytotoxic activity of samples obtained in various sage samples.

### 2.7 ANN modelling

A multi-layer perceptron model (MLP), employing three layers was used for artificial neural network (ANN) modelling, in order to explore the antioxidant activity of sage samples, according to the bioactive compounds content. The experimental database was normalized to enhance the conduct of the ANN model. The Broyden-Fletcher-Goldfarb-Shanno (BFGS) algorithm was engaged in solving nonlinear problems throughout the network modelling (Kollo & von Rosen, 2005). A sequence of various topologies (more than 100,000) were investigated in the course of ANN modelling, altering the number of neurons in the hidden layer (from 5 to 20), arbitrarily setting initial weights and biases (Ochoa-Martínez & Ayala-Aponte, 2007).

### 2.8 The accuracy of the model

The quantitative study of the erected ANN model's exactness was conducted according to the basic statistical tests, such as the coefficient of determination (*r*^2^), reduced chi-square (χ^2^), mean bias error (MBE),root mean square error (RMSE), mean percentage error (MPE), average absolute relative deviation (AARD) and sum of squared errors (SSE) (Aćimović et al., 2020).

### 2.9 Global sensitivity analysis

The Yoon’s interpretation method was exploited to ascertain the relative influence of bioactive compounds content on antioxidant activity of sage samples. This method was established using the weight coefficients of the developed ANN model (Yoon et al., 1993).

## 3 Results and discussion

### 3.1 Chemical profile

Chemical profile of analyzed EOs are given in Tables 1 (relative percentages) and 1S (quantitative profile, Supplementary material). Results showed that sesquiterpene viridiflorol is the principal compound in all six samples (17.68–33.08%), with the highest level in MWD 400 W. Besides viridiflorol, several other compounds were also detected in significant levels. Those were diterpene verticiol (6.80–13.50%), and monoterpenes camphor (8.60–19.50%), borneol (6.73–10.36%), α-thujone (3.50–12.20%), β-thujone (3.70–4.60%), and eucalyptol (0.43–8.94%). All these compounds are oxygenated terpenes with different functional groups. Viridiflorol and verticiol are alcohols with 15 and 20 carbon atoms (sesquiterpenoid and diterpenoid). Both were achieved their maximal content in MWD 400 W other three major compounds are monoterpenes with a different functional group. Eucalyptol is bicyclic ether; borneol is alcohol, while camphor and both thujones are ketones. Borneol and camphor have the same bicyclic structure where borneol may be easily converted to camphor by simple oxidation. The highest level of eucalyptol was achieved in MWD 800 W. The highest level of borneol was detected in MWD 200 W, while of camphor was in D 200 W. Same trend was noticed in the case of thujones, where maximal levels were observed in D 200 W. Both α-caryophyllene (1.44–2.44%) and β-caryophyllene (0.90–1.60%) were found in all EO samples, where α-caryophyllene was presented in higher levels in all six samples.

**Table1:** Chemical profile of sage essential oil samples.

Code	Name	Relative content (%)					
		D 200 W	D 400 W	MWD 200 W	MWD 400 W	MWD 600 W	MWD 800 W
1	α-Pinene	0.18 ± 0.01^d^	0.40 ± 0.02^e^	0.01 ± 0.00^a^	0.05 ± 0.00^c^	0.04 ± 0.00^b^	1.15 ± 0.09^f^
2	Camphene	0.07 ± 0.00^b^	1.30 ± 0.06^d^	0.04 ± 0.00^a^	ND	0.18 ± 0.01^c^	2.33 ± 0.11^e^
3	β-Pinene	ND**	ND	ND	ND	ND	0.07 ± 0.00
4	Myrcene	0.18 ± 0.01^d^	0.03 ± 0.00^c^	0.01 ± 0.00^a^	0.01 ± 0.00^a^	0.02 ± 0.00^b^	0.20 ± 0.10^e^
5	Limonene	0.10 ± 0.00^b^	0.25 ± 0.02^d^	0.03 ± 0.00^a^	0.03 ± 0.00^a^	0.15 ± 0.01^c^	0.90 ± 0.05^e^
6	Eucalyptol	6.93 ± 0.25^d^	8.25 ± 0.16^e^	3.54 ± 0.16^b^	0.43 ± 0.02^a^	4.32 ± 0.16^c^	8.94 ± 0.20^f^
7	*p*-Cymene	0.11 ± 0.01^c^	0.30 ± 0.01^e^	0.04 ± 0.00^b^	0.01 ± 0.00^a^	0.20 ± 0.01^d^	1.01 ± 0.03^f^
8	Fenchone	0.01 ± 0.00^a^	0.01 ± 0.00^a^	0.01 ± 0.00^a^	0.01 ± 0.00^a^	0.01 ± 0.00^a^	0.01 ± 0.00^a^
9	α-Thujone	12.20 ± 0.31^e^	11.70 ± 0.41^d^	11.05 ± 0.16^c^	3.50 ± 0.11^a^	10.60 ± 0.25^b^	11.80 ± 0.56^d^
10	β-Thujone	4.60 ± 0.13^e^	4.01 ± 0.14^d^	3.30 ± 0.09^b^	1.21 ± 0.10^a^	3.70 ± 0.12^c^	3.70 ± 0.15^c^
11	Menthone	0.03 ± 0.00^b^	0.03 ± 0.00^b^	0.03 ± 0.00^b^	0.02 ± 0.00^a^	0.03 ± 0.00^b^	0.03 ± 0.00^b^
12	*cis*-Linalool oxide	0.09 ± 0.01^c^	0.08 ± 0.01^c^	0.05 ± 0.00^a^	0.05 ± 0.00^a^	0.07 ± 0.00^b^	0.22 ± 0.01^d^
13	Camphor	19.50 ± 0.22^f^	16.20 ± 0.20^d^	18.10 ± 0.22^e^	8.60 ± 0.15^a^	14.30 ± 0.13^b^	15.01 ± 0.11^c^
14	Linalool	0.70 ± 0.03^b^	0.60 ± 0.02^a^	1.20 ± 0.06^e^	0.90 ± 0.03^c^	0.70 ± 0.03^b^	1.05 ± 0.05^d^
15	Linalyl acetate	ND	ND	0.02 ± 0.00^a^	0.03 ± 0.00^b^	0.03 ± 0.00^b^	0.30 ± 0.01^c^
16	Bornyl acetate	2.78 ± 0.06^c^	2.58 ± 0.05^b^	2.87 ± 0.09^c^	2.90 ± 0.10^c^	2.69 ± 0.11^b^	2.37 ± 0.11^a^
17	*trans*-β-Caryophyllene	1.20 ± 0.05^c^	1.10 ± 0.03^b^	0.90 ± 0.02^a^	1.10 ± 0.03^b^	1.50 ± 0.02^d^	1.60 ± 0.03^e^
18	Terpinen-4-ol	0.35 ± 0.01^b^	0.41 ± 0.01^c^	0.35 ± 0.01^b^	0.30 ± 0.01^a^	0.42 ± 0.01^c^	0.60 ± 0.02^d^
19	Menthol	0.28 ± 0.01^b^	0.22 ± 0.01^a^	0.40 ± 0.02^d^	0.44 ± 0.01^e^	0.30 ± 0.01^c^	0.28 ± 0.01^b^
20	Alloaromadendrene	ND	ND	0.12 ± 0.01^a^	0.16 ± 0.01^b^	0.33 ± 0.01^c^	0.32 ± 0.01^c^
21	Isothujol	0.05 ± 0.01^b^	0.05 ± 0.00^b^	0.08 ± 0.00^c^	0.08 ± 0.00^c^	0.05 ± 0.00^b^	0.04 ± 0.00^a^
22	α-Caryophyllene	1.81 ± 0.09^b^	1.99 ± 0.05^b^	1.44 ± 0.09^a^	1.78 ± 0.08^b^	2.44 ± 0.11^c^	2.35 ± 0.12^c^
23	Ledene	0.44 ± 0.03^c^	0.45 ± 0.02^c^	0.42 ± 0.01^b^	0.34 ± 0.01^a^	0.66 ± 0.03^e^	0.55 ± 0.10^d^
24	Borneol	8.85 ± 0.03^c^	6.73 ± 0.03^b^	10.36 ± 0.18^e^	9.59 ± 0.16^d^	6.90 ± 0.18^b^	6.30 ± 0.22^a^
25	Carvyl acetate	0.02 ± 0.00^b^	0.03 ± 0.00^c^	0.03 ± 0.00^c^	0.02 ± 0.00^b^	0.02 ± 0.00^b^	0.01 ± 0.00^a^
26	γ-Muurolen	0.10 ± 0.01^c^	0.10 ± 0.01^c^	0.07 ± 0.00^a^	0.10 ± 0.01^c^	0.09 ± 0.01^b^	0.11 ± 0.01^c^
27	Carane	0.07 ± 0.00^a^	0.09 ± 0.01^b^	0.15 ± 0.01^e^	0.13 ± 0.01^d^	0.11 ± 0.01^c^	0.50 ± 0.01^f^
28	Myrthenol	0.20 ± 0.01^d^	0.18 ± 0.01^c^	0.22 ± 0.01^e^	0.23 ± 0.01^e^	0.16 ± 0.01^b^	0.12 ± 0.01^a^
29	Dehydroaromadendrene	0.21 ± 0.01^b^	0.22 ± 0.01^b^	0.11 ± 0.01^a^	0.21 ± 0.01^b^	0.25 ± 0.01^c^	0.22 ± 0.01^b^
30	Spathulenol	0.44 ± 0.03^a^	0.49 ± 0.02^b^	0.50 ± 0.03^b^	0.71 ± 0.03^e^	0.66 ± 0.02^d^	0.52 ± 0.01^c^
31	*cis*-Carveol	0.09 ± 0.01^a^	0.12 ± 0.01^b^	0.15 ± 0.01^c^	0.15 ± 0.01^c^	0.11 ± 0.01^b^	0.09 ± 0.01^a^
32	Geraniol	0.08 ± 0.00^b^	0.06 ± 0.00^a^	0.20 ± 0.01^e^	0.15 ± 0.01^d^	0.09 ± 0.01^b^	0.12 ± 0.01^c^
33	*p*-Cymen-8-ol	0.15 ± 0.01^a^	0.18 ± 0.01^b^	0.15 ± 0.01^a^	0.19 ± 0.01^b^	0.18 ± 0.01^b^	0.22 ± 0.01^c^
34	Phenylethyl alcohol	ND	ND	0.66 ± 0.02^a^	1.10 ± 0.09^b^	0.99 ± 0.05^b^	0.62 ± 0.03^a^
35	Caryophyllene oxide	1.20 ± 0.05^b^	1.14 ± 0.03^b^	1.22 ± 0.04^b^	1.62 ± 0.08^c^	1.14 ± 0.04^b^	0.89 ± 0.04^a^
36	Globulol	ND	ND	0.20 ± 0.01^c^	0.28 ± 0.01^d^	0.15 ± 0.01^b^	0.11 ± 0.01^a^
37	Ledol	0.15 ± 0.01^b^	0.12 ± 0.01^a^	1.15 ± 0.03^d^	1.33 ± 0.02^e^	1.20 ± 0.05^d^	0.90 ± 0.02^c^
38	Caryophyllenyl alcohol	0.77 ± 0.04^a^	0.72 ± 0.03^a^	ND	ND	ND	ND
39	Viridiflorol	20.26 ± 0.62^b^	21.66 ± 0.55^b^	23.09 ± 0.48^c^	33.08 ± 0.60^d^	23.07 ± 0.36^c^	17.68 ± 0.16^a^
40	Aromadendrene oxide	0.08 ± 0.01^a^	0.07 ± 0.01^a^	5.15 ± 0.11^c^	8.22 ± 0.12^e^	5.89 ± 0.10^d^	4.31 ± 0.12^b^
41	Widdrol	0.21 ± 0.01^a^	0.23 ± 0.02^a^	ND	ND	ND	ND
42	*o*-Cymen-5-ol	0.29 ± 0.01^a^	0.41 ± 0.02^b^	ND	ND	ND	ND
43	Thymol	1.10 ± 0.06^c^	0.95 ± 0.03^b^	1.21 ± 0.09^d^	1.52 ± 0.08^e^	0.99 ± 0.03^b^	0.65 ± 0.02^a^
44	Carvacrol	0.52 ± 0.03^b^	0.44 ± 0.02^a^	0.77 ± 0.02^c^	0.82 ± 0.03^d^	0.55 ± 0.04^b^	0.52 ± 0.03^b^
45	Ledene oxide-(II)	4.20 ± 0.12^a^	4.30 ± 0.15^a^	ND	ND	ND	ND
46	Isoaromadendrene epoxide	2.10 ± 0.09^b^	2.40 ± 0.09^c^	2.50 ± 0.12^c^	3.90 ± 0.11^d^	2.70 ± 0.09^c^	1.70 ± 0.05^a^
47	Verticiol	6.80 ± 0.16^a^	8.70 ± 0.20^c^	7.50 ± 0.06^b^	13.50 ± 0.12^e^	11.00 ± 0.21^d^	8.70 ± 0.12^c^
48	Valencene	0.50 ± 0.01 ^a^	0.70 ± 0.01^c^	0.60 ± 0.01^b^	1.20 ± 0.02^e^	1.01 ± 0.03^d^	0.70 ± 0.01^c^

*Means in the same row with different superscript are statistically different (*p* ≤ 0.05), according to post - doc Tukey's HSD test.

**ND-not detected.

Quantitative profile (Table 1S, Supplementary material) demonstrated similar situation like in the case of relative content. High content of camphor (55.01–144.09 mg/g), eucalyptol (5.29–79.26 mg/g), α-thujone (8.97–85.48 mg/g), β-thujone (2.89–29.70%), and borneol (45.80–92.45 mg/g) were noticed. The same trends were noticed in the case of the influence of the extraction parameters. All ketones achieved maximal yield in D 200 W, the yield of eucalyptol reached its maximum in MWD 800 W, while borneol was in MWD 200 W. When comparing the yields of alcohols borneol, and linalool with the yields of their less polar derivatives borneol acetate and linalool acetate, different behavior might be noticed. Borneol and bornyl acetate were achieved their maximal yield in the same sample (MWD 200 W), while situation was slightly different in the case of linalool and linalyl acetate. The highest yield of linalool was in sample MWD 200 W, while linalyl acetate was not found in samples obtained by hydrodistillation, while the highest yield achieved in MWD 800 W. Although linalool is hydrocarbon alcohol it achieved its maximal yield at same condition as borneol. On the other hand, structural differences obviously showed much higher influence in the case of their acetate derivatives.

**Fig. 1:**
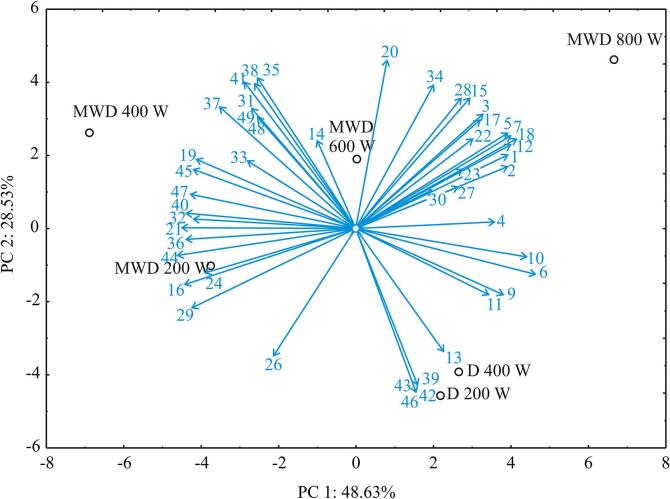
The PCA biplot diagram, depicting the relationships among bioactive compounds content in sage samples. The shown compound codes (1–49) were explained in Table 1.

In order to thoroughly describe the structure of the exploratory data that would provide a better perception of dissimilarities between different sage samples, according to the bioactive compounds content, PCA was used, and the results are displayed in Fig. 1. The first two PCs explained 77.16% of the total variance in the experimental data. According to results, sample MWD 400 W was characterized by the increased amount of bioactive compounds such as: bornyl acetate, menthol, isothujol, myrthenol, spathulenol, *cis*-carveol, phenylethyl alcohol, caryophyllene oxide, globulol, ledol, viridiflorol, aromadendrene oxide, thymol, verticiol, and valencene, while sample MWD 800 W was described by the augmented concentration of following bioactive compounds: α-pinene, camphene, β-pinene, limonene, eucalyptol, p-cymene, α-thujone, β-thujone, *cis*-linalool oxide, linalyl acetate, *trans*-β-caryophyllene, terpinen-4-ol, alloaromadendrene, carane, and *p*-cymen-8-ol. It might be seen that mentioned compounds in MWD 400 W sample are oxygenated terpenes, while majority of above-mentioned compounds in sample MWD 800 W are monoterpenes. Samples D 200 W and D 400 W were classified according to the increased concentration of bioactive compounds like: camphor, caryophyllenyl alcohol, widdrol, *o*-cymen-5-ol and ledene oxide-(II). Linalyl acetate was not detected in MWD samples, while β-pinene was found in MWD-800 W.

### 3.2 Thermal analysis

**Fig. 2:**
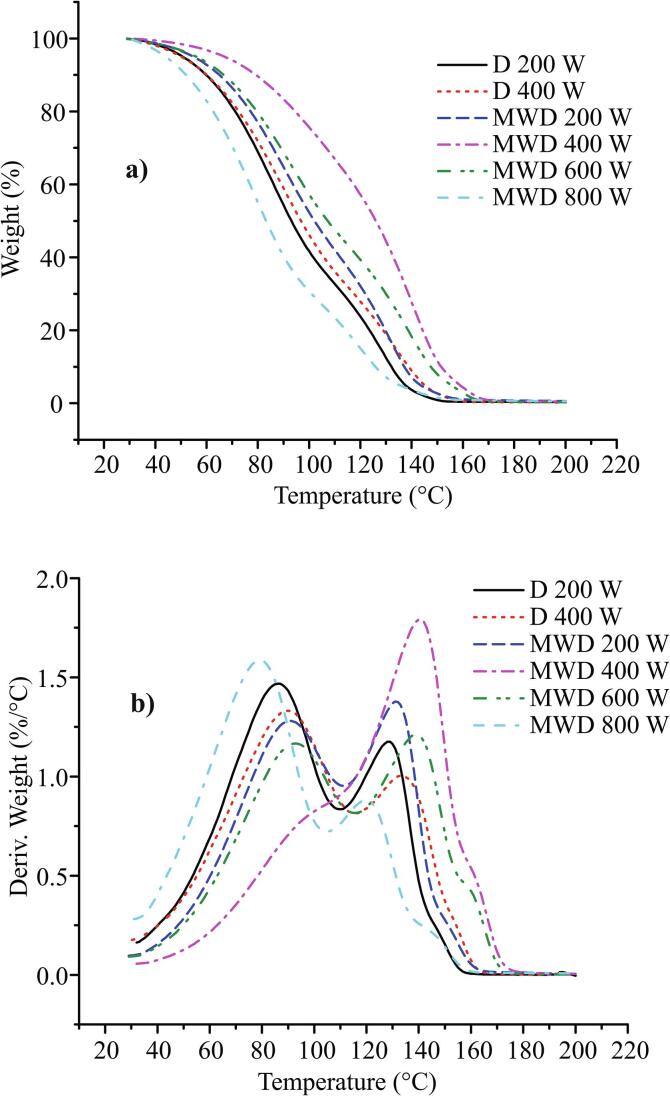
a) Thermogravimetric (TG) and b) differential thermogravimetric (dTG) curves of the evaporation process of sage essential oils obtained under a different extraction conditions. Heat rate 5 °C/min, nitrogen purge flow 60 mL/min.

The evaporation process of tested sage EOs was analyzed using thermogravimetry, in non-isothermal conditions. The obtained thermogravimetric (TG) and differential thermogravimetric (dTG) curves are shown in Fig. 2.

The evaporation process of all samples started at room temperature and ended at about 153 °C for samples D 200 W and MWD 800 W, about 158 °C for samples D 400 W and MWD 200 W, and about 167 °C for samples MWD 400 W and MWD 600 W. It can be seen that the evaporation rates significantly differed between the samples from the TG curves. The MWD 800 and MWD 400 samples were two extremes; the MWD 800 had the highest evaporation rate, and the MWD 400 the lowest. The evaporation rates of the rest of the samples were between these two extreme cases. Two peaks and one shoulder at the end of the evaporation process were detected on all dTG curves, except for the MVD 400 sample. One peak with a larger shoulder on the left and a smaller shoulder on the right side was detected on the dTG curve of the MWD 400 sample. These facts indicate that the evaporation process of analyzed EOs is a complex one. This could be expected, since essential oils are mixtures of a large number of components with different volatility. Almost 50 components were detected in these EOs (Table 1S, Supplementary material). Two peaks on dTG curves point out that the evaporation process took place in two steps. The first one, where more volatile components evaporated, and the second one, where residual, less volatile components, evaporated. The boiling point of components can be taken as one of the measures of their volatility. The most prevalent components (make up greater than 80% of the total share in the samples, Table 1) can be divided into two groups: more volatile (MV) (Camphor, α-Thujone, β-Thujone, Borneol, Eucalyptol, Bornyl acetate, Camphene, α-Caryophyllene) - with boiling points of about 160 to 220 °C (ChemSpider |Search and Share Chemistry, n.d.), and less volatile (LV) (Viridiflorol, Verticiol, Ledene oxide-(II), Aromadendrene oxide, Isoaromadendrene epoxide) - with boiling points of about 270 to 380 °C (*ChemSpider | Search and Share Chemistry*, n.d.). Based on the results in Table 1, the ratio of MV/LV components can be calculated and it was as follows per the samples: D 200 W – 54/35, D 400 W – 49/37, MWD 200 W – 49/38, MWD 400 W – 25/59, MWD 600 W – 43/43 and MWD 800 W – 54/31. All samples, except MWD 400 and 600 W, had a significantly higher proportion of MV components compared to LV ones. The MWD 600 W sample had approximately the equal ratio, while the MWD 400 W had a significantly higher share of LV components. This ratio of MV and LV components in the samples can serve to explain the shape of the obtained TG and DTG curves. In the case of the samples with a higher or equal share of MV components compared to LV components (all samples, except MWD 400 W), the effect of evaporation of MV components was dominant at lower temperatures. As a consequence, the first peak on the dTG curves arose. As the temperature increased, LV components accumulated in the samples. At higher temperatures, the effect of their evaporation became dominant, resulting in the appearance of a second peak on the dTG curves. In the case of MWD 400 W sample, due to the significantly higher share of LV components, their evaporation effect at lower temperatures was not negligible as in the other samples. As a result of the overlapping effects of the evaporation of MV and LV components, the shoulder appeared on the dTG curve at lower temperatures. At higher temperatures, a peak on dTG curve stood out, as a consequence of evaporation of residual LV components in the sample. Also, a large proportion of LV components was the cause of the lowest evaporation rate of MWD 400 W sample.

### 3.3 Biological activity of essential oil samples

**Fig. 3:**
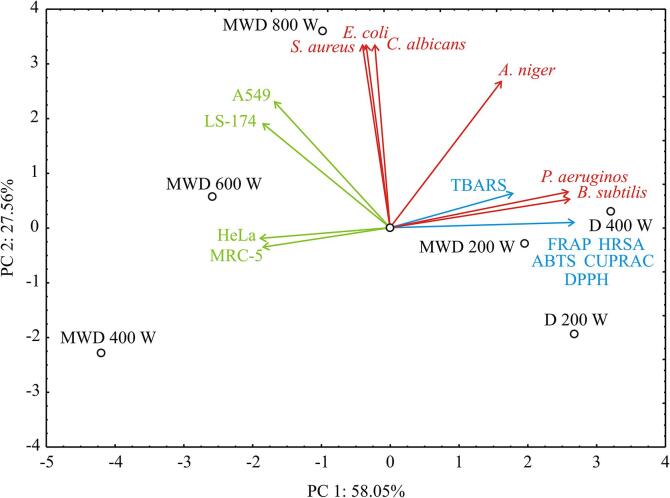
The PCA biplot diagram, depicting the relationships among antioxidant activity, microbiological data and cytotoxic activity of samples.

Results of antioxidant, antimicrobial, and cytotoxic activity assessments are given in Tables 2S, 3S, and 4S, respectively. To thoroughly describe the structure of the exploratory data that would provide a better perception of dissimilarities between different sage samples, according to the antioxidant activity, microbiological data and cytotoxic activity of samples, PCA was used, and the results are displayed in Fig. 3. The first two PCs explained 86.36% of the total variance in the experimental data. All antioxidant activity assays (Table 2S) showed that MWD 400 W was the most potent sample. On the other hand, D 200 W was the least potent sample in the most cases. Sample MWD 400 W showed the highest content of viridiflorol, caryophyllene oxide, aromadendrene oxide, thymol, carvacrol, and verticiol. Some of these compounds or their synergistic activity may be the explanation for such potency of this sample. Antimicrobial activity (Table 3S) showed somewhat different activity. D 200 W showed highest activity against *Staphylococcus aureus, Escherichia coli*, and *Candida albicans*. On the other hand, MWD 400 W showed the highest activity against *Bacillus subtilis*, *Pseudomonas aeruginosa*, and *Aspergillus niger*. MTT assay also revealed diversity in activity of the tested samples. D 200 W was the most potent against MRC-5 cell line, while D 400 W was the most potent against other tested cell lines (HeLa, LS-174, and A549).

**Fig. 4:**
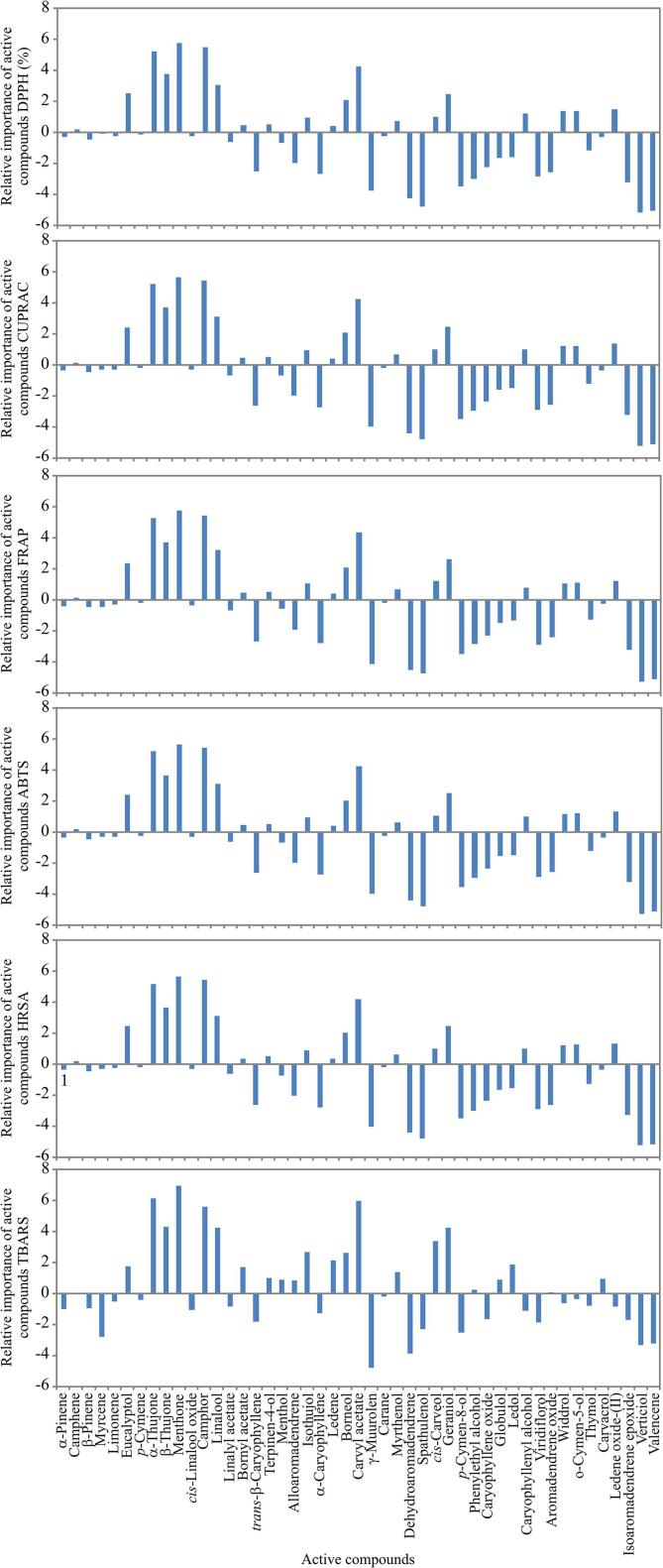
The relative importance of the bioactive compounds content on antioxidant assays, determined using Yoon interpretation method.

Previous studies showed that biological activity is closely related to the chemical composition of the tested samples (Table1, Table1S, chromatograms are given in Figures 1-6S, Supplementary material). Bosnić et al. (2006) have determined the 1,8-cineole compound appears to be more effective against gram-positive strains (*S. aureus* and *B. subtillis*) (Bosnić et al., 2006). In other words, the presence or absence of the compounds with a certain functional group may significantly impact the activity (Riabov et al., 2020). Thus, compounds such as α-pinene, 1,8-cineole (eucalyptol), p-cymene, menthol, and terpinene-4-ol showed insignificant activity against DPPH radicals. This study also showed that α-terpineol and α-phellandrene showed moderate activity, while γ-terpinene, pulegone, myrcene, citral, and carvone showed significant activity (Wojtunik et al., 2014). Research also showed that the presence of the double bond in the structure enhances the activity, while molecules with conjugated double bonds neutralize radicals very quickly (Riabov et al., 2020). The higher potency of the 1,4-cyclohexadiene system against free radicals is the reason why γ-terpinene is more potent than α-terpinene. However, the aromatic system decreases the activity, but the hydroxyl functional group in fact increases the antioxidant activity of the molecules. The relationship between the structure and activity might be clearly seen in the case of the comparison of the activity of p-cymene, thymol, menthone, menthol, and pulegone (Wojtunik et al., 2014).

On the other hand, the ability to make hydrogen bonds is proven to be very important for antimicrobial activity (Griffin et al., 1999). Griffin et al. (1999) also reported that geometric isomerism did not affect the activity by testing nerol and geraniol. They also reported that the position of the functional group does not influence the activity. However, the presence/absence of the hydroxyl functional group in the structure is of great importance to antimicrobial activity (Griffin et al., 1999). Previous studies showed that alcohol was more active than appropriate aldehyde/ketone (Trombetta et al., 2005; Zengin & Baysal, 2014). Besides the hydroxyl functional group, the presence and position of the double bond are also important for the activity (Riabov et al., 2020). Thus, terpinene-4-ol, in this case, was more potent than α-terpineol, which was not the case for antioxidant activity. The explanation for the higher potency of terpinene-4-ol is the position of the OH functional group, which makes this molecule more suitable for making hydrogen bonds (Griffin et al., 1999). Since sage is rich in essential oils, its antimicrobial potential has a wide variability depending on the sensitivity of microorganisms and the efficiency of the tested compounds. It was also stated that hydrophobicity/lipophobicity also had an important role in determining antimicrobial potency. They divide into a lipid bilayer of the cell membrane, making it more permeable, causing leakage of vital cell contents (Beheshti-Rouy et al., 2015). Differences in these properties make these molecules easily permeable through the cell membrane, thus interfering with the fluidity and permeability of the membrane and leading to the cell lysis (Andrade-Ochoa et al., 2015, Zengin and Baysal, 2014).

### 3.4 ANN model

The obtained optimal neural network model could be applied to adequately anticipate the antioxidant activity of sage samples, on the basis of bioactive compounds content. The optimal number of neurons in the hidden layer was 9 (network MLP 47–9-6), while the highest *r*^2^ values during the training cycle were 0.998).

The obtained ANN model for the prediction of outputs was complex (492 weights-biases coefficients) due to the high nonlinear relation between variables in the database. The goodness of fit tests results between experimental and ANN model were shown in Table 5S.

The ANN predicted values were in a close viscinity to the measured values in most cases, in terms of r2 values (Erbay and Icier, 2009, Turányi and Tomlin, 2014). The SSE values obtained with the ANN model was of the same order of magnitude as experimental errors for output variables presented in many references (Doumpos & Zopounidis, 2011). The ANN model had an insignificant lack of fit tests, which means the model satisfactorily predicted output variables.

#### Global sensitivity analysis- Yoon’s interpretation method

According to the Fig. 4, *α*-thujone and menthone content showed the stronger positive influence on DPPH, CUPRAC, FRAP, ABTS and HRSA, while the content of verticiol and valencene content exerted the negative influence on DPPH, CUPRAC, FRAP, ABTS and HRSA. The positive influence on TBARS was observed for *α*-thujone, menthone, camphor and carvyl acetate content.

These conclusions are in line with PCA results, in which the highest content of *α*-thujone and menthone (codes 9 and 11, respectively, according to Fig. 1) were obtained for samples D 400 W and D 200 W, while the most pronounced antioxidant capacity was observed for samples D 200 W and D 400 W (Fig. 3).

## 4 Conclusion

This study showed that procedure of the EOs preparation may significantly influence the chemical composition, thermal behaviour, and biological activity of the essential oils. Although same compound was the principal in all samples (viridiflorol), content and presence/absence of the minor compounds was significantly different. Besides viridiflorol, several other compounds such as 1,8-cineole (eucalyptol), α- and β-thujones, camphor, borneol, and verticiol were also found in significant amounts. Diversities in the composition induced the diversities in the biological activity, where MWD 400 W was the most potent antioxidant agent. On the other hand, in the case of antimicrobial and cytotoxic activities situation was slightly different. Structural diversities influenced in the way that D 200 W and MWD 400 W were the most potent antibacterial agents depending on the microbial strain. In the case of cytotoxic activity, samples prepared by classical hydrodistillation (D 200 W and D 400 W) were the most potent cytotoxic agents.

The artificial neural network model expressed quite a good fit for anticipating antioxidant assays according to the bioactive content, (*r^2^* values during training cycle for these variables were: 0.998). According to the Yoon interpretation method, *α*-thujone and menthone content expressed the highest positive effect on DPPH, CUPRAC, FRAP, ABTS and HRSA, while the content of verticiol and valencene content showed the stronger negative influence on DPPH, CUPRAC, FRAP, ABTS and HRSA. The positive influence on TBARS was observed for *α*-thujone, menthone, camphor and carvyl acetate content.

## Declaration of Competing Interest

The authors declare that they have no known competing financial interests or personal relationships that could have appeared to influence the work reported in this paper.
