# Supplementary material for: The effect of various extraction techniques on the quality of sage (Salvia officinalis L.) essential oil, expressed by chemical composition, thermal properties and biological activity
Source: Food Chem X. 2022 Jan 19;13:100213. doi: 10.1016/j.fochx.2022.100213 (PMC9039893; doi:10.1016/j.fochx.2022.100213)
Supplement: Supplementary Data 1 [file mmc1.docx]

The effect of various extraction techniques on the quality of sage (*Salvia officinalis* L.) essential oil, expressed by chemical composition, thermal properties and biological activity

Saša Đurović^a,^*, Darko Micić^a^, Lato Pezo^a^, Danka Radić^a^, Julia G. Bazarnova^b^, Yulia A. Smyatskaya^b^, Stevan Blagojević^a^

^a^Institute of General and Physical Chemistry, Studentski trg 12, 11158 Belgrade, Serbia

^b^Peter the Great Saint-Petersburg Polytechnic University, Graduate School of Biotechnology and food industries, Polytechnicheskaya street, 29, 195251 Saint-Petersburg, Russia

*Laboratory of Chromatography, Institute of General and Physical Chemistry, Studentski trg 12/V, 11158 Belgrade, Republic of Serbia, Tel: +381659577200, e-mail: [sasatfns@uns.ac.rs](mailto:sasatfns@uns.ac.rs).

Table 1S. Quantitative profile of sage essential oil samples

| Name | Content (mg/g) | | | | | |
| --- | --- | --- | --- | --- | --- | --- |
|  | D 200 W | D 400 W | MWD 200 W | MWD 400 W | MWD 600 W | MWD 800 W |
| α-Pinene | 0.25 ± 0.03^c^ | 3.24 ± 0.09^d^ | 0.01 ± 0.00^a^ | 0.01 ± 0.00^a^ | 0.02 ± 0.00^b^ | 10.39 ± 0.02^e^ |
| Myrcene | 0.12 ± 0.01^c^ | 0.38 ± 0.01^e^ | 0.03 ± 0.00^b^ | 0.01 ± 0.00^a^ | 0.15 ± 0.00^d^ | 3.24 ± 0.02^f^ |
| Limonene | 0.28 ± 0.01^c^ | 1.06 ± 0.02^e^ | 0.15 ± 0.01^b^ | 0.06 ± 0.00^a^ | 0.37 ± 0.01^d^ | 9.34 ± 0.04^f^ |
| Eucalyptol | 50.14 ± 0.08^d^ | 69.89 ± 0.38^e^ | 25.74 ± 0.09^b^ | 5.29 ± 0.05^a^ | 37.13 ± 0.41^c^ | 79.26 ± 0.55^f^ |
| *p*-Cymene | 0.28 ± 0.01^c^ | 1.05 ± 0.01^e^ | 0.06 ± 0.00^b^ | 0.04 ± 0.00^a^ | 0.40 ± 0.01^d^ | 6.65 ± 0.06^f^ |
| Fenchone | 0.01 ± 0.00^a^ | 0.01 ± 0.00^a^ | 0.01 ± 0.00^a^ | 0.01 ± 0.00^a^ | 0.01 ± 0.00^a^ | 0.01 ± 0.00^a^ |
| α-Thujone | 85.48 ± 0.44^f^ | 56.35 ± 0.32^d^ | 35.12 ± 0.16^b^ | 8.97 ± 0.06^a^ | 53.32 ± 0.26^c^ | 64.74 ± 0.34^e^ |
| β-Thujone | 29.70 ± 0.22^f^ | 20.97 ± 0.19^d^ | 10.00 ± 0.05^b^ | 2.89 ± 0.03^a^ | 20.43 ± 0.19^c^ | 27.17 ± 0.21^e^ |
| Menthone | 0.09 ± 0.00^b^ | 0.14 ± 0.00^c^ | 0.10 ± 0.00^b^ | 0.04 ± 0.00^a^ | 0.09 ± 0.01^b^ | 0.09 ± 0.00^b^ |
| Camphor | 144.09 ± 0.70^e^ | 141.08 ± 0.04^d^ | 140.54 ± 0.47^d^ | 55.01 ± 0.37^a^ | 116.99 ± 0.63^b^ | 123.38 ± 0.75^c^ |
| Linalool | 11.81 ± 0.02^e^ | 11.16 ± 0.02^c^ | 16.43 ± 0.09^f^ | 8.10 ± 0.14^a^ | 8.76 ± 0.02^b^ | 11.66 ± 0.07^d^ |
| Linalyl acetate | ND** | ND | 0.02 ± 0.00^a^ | 0.02 ± 0.00^a^ | 0.04 ± 0.00^b^ | 3.66 ± 0.03^c^ |
| Bornyl acetate | 22.87 ± 0.06^c^ | 24.30 ± 0.10^e^ | 24.78 ± 0.10^f^ | 19.46 ± 0.28^a^ | 23.83 ± 0.17^d^ | 20.34 ± 0.11^b^ |
| *trans*-β-Caryophyllene | 10.93 ± 0.05^b^ | 12.89 ± 0.07^c^ | 7.35 ± 0.01^a^ | 7.44 ± 0.15^a^ | 14.20 ± 0.10^d^ | 14.39 ± 0.15^d^ |
| Terpinen-4-ol | 5.52 ± 0.04^e^ | 3.07 ± 0.03^c^ | 2.06 ± 0.03^b^ | 1.10 ± 0.01^a^ | 3.72 ± 0.05^d^ | 3.00 ± 0.04^c^ |
| Menthol | 2.86 ± 0.06^c^ | 2.86 ± 0.13^c^ | 4.53 ± 0.04^e^ | 4.12 ± 0.06^d^ | 2.43 ± 0.07^b^ | 1.69 ± 0.06^a^ |
| Borneol | 65.63 ± 0.24^d^ | 58.64 ± 0.24^c^ | 90.45 ± 0.25^f^ | 69.54 ± 1.01^e^ | 55.68 ± 0.06^b^ | 45.80 ± 0.22^a^ |
| Caryophyllene oxide | 12.58 ± 0.06^e^ | 4.31 ± 0.02^b^ | 4.63 ± 0.03^c^ | 4.65 ± 0.02^c^ | 5.43 ± 0.03^d^ | 3.57 ± 0.01^a^ |
| Thymol | 4.90 ± 0.15^b^ | 5.20 ± 0.09^c^ | 9.36 ± 0.11^e^ | 8.02 ± 0.04^d^ | 4.73 ± 0.10^b^ | 1.95 ± 0.00^a^ |
| Carvacrol | 2.76 ± 0.03^c^ | 2.84 ± 0.02^d^ | 5.34 ± 0.10^f^ | 5.10 ± 0.13^e^ | 2.49 ± 0.03^b^ | 0.93 ± 0.06^a^ |

^*^Means in the same row with different superscript are statistically different (*p* ≤ 0.05), according to post - doc Tukey's HSD test.

**ND-not detected.

Table 2S. Antioxidant activity of sage samples

| Sample | IC_50_ (µg/mL) | | | | | |
| --- | --- | --- | --- | --- | --- | --- |
|  | DPPH | CUPRAC | FRAP | ABTS | HRSA | TBARS |
| D 200 W | 2.34 ± 0.01^e^ | 4.26 ± 0.03^f^ | 3.20 ± 0.03^e^ | 6.39 ±0.03^f^ | 5.33 ± 0.06^f^ | 12.16 ± 0.03^d^ |
| D 400 W | 2.17 ± 0.02^d^ | 3.94 ± 0.02^d^ | 2.96 ± 0.02^d^ | 5.91 ± 0.05^d^ | 4.93 ± 0.03^d^ | 12.22 ± 0.06^d^ |
| MWD 200 W | 2.20 ± 0.03^d^ | 4.12 ± 0.05^e^ | 3.25 ± 0.01^f^ | 6.22 ± 0.04^e^ | 5.15 ± 0.05^e^ | 20.32 ± 0.03^e^ |
| MWD 400 W | 0.41 ± 0.01^a^ | 0.74 ± 0.01^a^ | 0.56 ± 0.01^a^ | 1.11 ± 0.02^a^ | 0.93 ± 0.01^a^ | 5.26 ± 0.02^a^ |
| MWD 600 W | 1.02 ± 0.02^b^ | 1.86 ± 0.02^b^ | 1.40 ± 0.02^b^ | 2.79 ± 0.03^b^ | 2.32 ± 0.02^b^ | 11.26 ± 0.03^c^ |
| MWD 800 W | 1.40 ± 0.03^c^ | 2.54 ± 0.03^c^ | 1.91 ± 0.02^c^ | 3.81 ± 0.05^c^ | 3.18 ± 0.02^c^ | 11.15 ± 0.05^b^ |

^*^Means in the same column with different superscript are statistically different (*p* ≤ 0.05), according to post - doc Tukey's HSD test.

Table 3S. Microbiological data of sage samples

| Microbial strain | Disc diffusion method (15 mL of EO concentration 100%) | | | | | | | | |
| --- | --- | --- | --- | --- | --- | --- | --- | --- | --- |
|  | D 200 W | D 400 W | MWD 200 W | MWD 400 W | MWD 600 W | MWD 800 W | Positive control | | Negative control |
|  |  |  |  |  |  |  | CHL | ACT | 5% DMSO |
| *Staphylococcus aureus* (ATCC 25923) | 21.42 ± 0.15^a^ | 26.18 ± 0.12^d^ | 23.56 ± 0.11^c^ | 22.14 ± 0.11^b^ | 27.06 ± 0.11^d^ | 32.47 ± 0.16^f^ | 29.67 ± 0.58^e^ | ND | ND |
| *Escherichia coli* (ATCC 25922) | 12.42 ± 0.09^a^ | 15.18 ± 0.09^d^ | 13.66 ± 0.16^c^ | 12.78 ± 0.09^b^ | 15.62 ± 0.09^e^ | 18.74 ± 0.11^f^ | 29.33 ± 1.53^g^ | ND | ND |
| *Bacillus subtilis* (ATCC 6633) | 14.94 ± 0.09^d^ | 18.26 ± 0.08^f^ | 16.43 ± 0.11^e^ | 9.00 ±0.05^a^ | 11.00 ± 0.08^b^ | 13.20 ± 0.09^c^ | 28.00 ± 1.00^g^ | ND | ND |
| *Pseudomonas aeruginosa* (ATCC 27853) | 19.08 ± 0.16^e^ | 23.32 ± 0.16^g^ | 20.98 ± 0.16^f^ | 11.88 ± 0.09^a^ | 14.52 ± 0.09^c^ | 17.42 ± 0.12^d^ | 12.33 ± 0.58^b^ | ND | ND |
| *Aspergillus niger* (ATCC 16404) | 21.60 ± 0.16^b^ | 26.40 ± 0.11^e^ | 23.76 ± 0.16^d^ | 18.00 ± 0.28^a^ | 22.00 ± 0.21^c^ | 26.40 ± 0.16^e^ | ND | 26.33 ± 0.58^e^ | ND |
| *Candida albicans* (ATCC 10231) | 18.18 ± 0.12^a^ | 22.22 ± 0.11^c^ | 19.99 ± 0.12^b^ | 18.36 ± 0.22^a^ | 22.44 ± 0.20^c^ | 26.92 ± 0.12^d^ | ND | 35.00 ± 0.02^e^ | ND |

^*^Means in the same row with different superscript are statistically different (*p* ≤ 0.05), according to post - doc Tukey's HSD test.

CHL-Chloramphenicol, ACT-Actidion, DMSO-dimethyl sulfoxide, ND-not detected.

Table 4S. Cytotoxic activity of sage samples

| Cell line | IC_50_ (µg/mL) | | | | | | |
| --- | --- | --- | --- | --- | --- | --- | --- |
|  | D 200 W | D 400 W | MWD 200 W | MWD 400 W | MWD 600 W | MWD 800 W | CDDP |
| HeLa | 90.00 ± 11.20^b^ | 75.33 ± 10.02^b^ | 222.03 ± 16.25^d^ | 218.34 ± 14.56^d^ | 208.71 ± 10.23^d^ | 154.17 ± 11.33^c^ | 2.34 ± 0.70^a^ |
| LS-174 | 206.25 ± 12.50^b^ | 177.87 ± 16.22^b^ | 390.50 ± 18.22^c^ | 373.12 ± 13.26^c^ | 538.34 ± 11.56^d^ | 534.49 ± 11.26^d^ | 10.05 ± 0.11^a^ |
| A549 | 296.73 ± 16.20^b^ | 271.36 ± 18.11^b^ | 364.82 ± 11.02^c^ | 387.09 ± 19.52^c^ | 414.36 ± 12.20^d^ | 522.55 ± 12.20^e^ | 5.16 ± 0.21^a^ |
| MRC-5 | 156.33 ± 18.20^b^ | 168.30 ± 11.63^b^ | 325.80 ± 12.20^d^ | 325.08 ± 11.52^d^ | 368.28 ± 16.63^e^ | 214.74 ± 11.23^c^ | 9.08 ± 0.90^a^ |

^*^Means in the same column with different superscript are statistically different (*p* ≤ 0.05), according to post - doc Tukey's HSD test

CDDP-cisplatin.

Table 5S. The "goodness of fit" tests for the developed ANN model

|  | χ^2^ | RMSE | MBE | MPE | SSE | AARD | r^2^ |
| --- | --- | --- | --- | --- | --- | --- | --- |
| DPPH | 0.006 | 0.071 | -0.033 | 2.013 | 0.024 | 0.235 | 0.994 |
| CUPRAC | 0.022 | 0.137 | -0.068 | 2.014 | 0.084 | 0.447 | 0.994 |
| FRAP | 0.017 | 0.120 | -0.056 | 2.134 | 0.068 | 0.363 | 0.991 |
| ABTS | 0.049 | 0.203 | -0.099 | 1.905 | 0.188 | 0.640 | 0.994 |
| HRSA | 0.035 | 0.172 | -0.086 | 2.017 | 0.133 | 0.564 | 0.994 |
| TBARS | 0.241 | 0.448 | -0.101 | 4.110 | 1.144 | 1.976 | 0.991 |


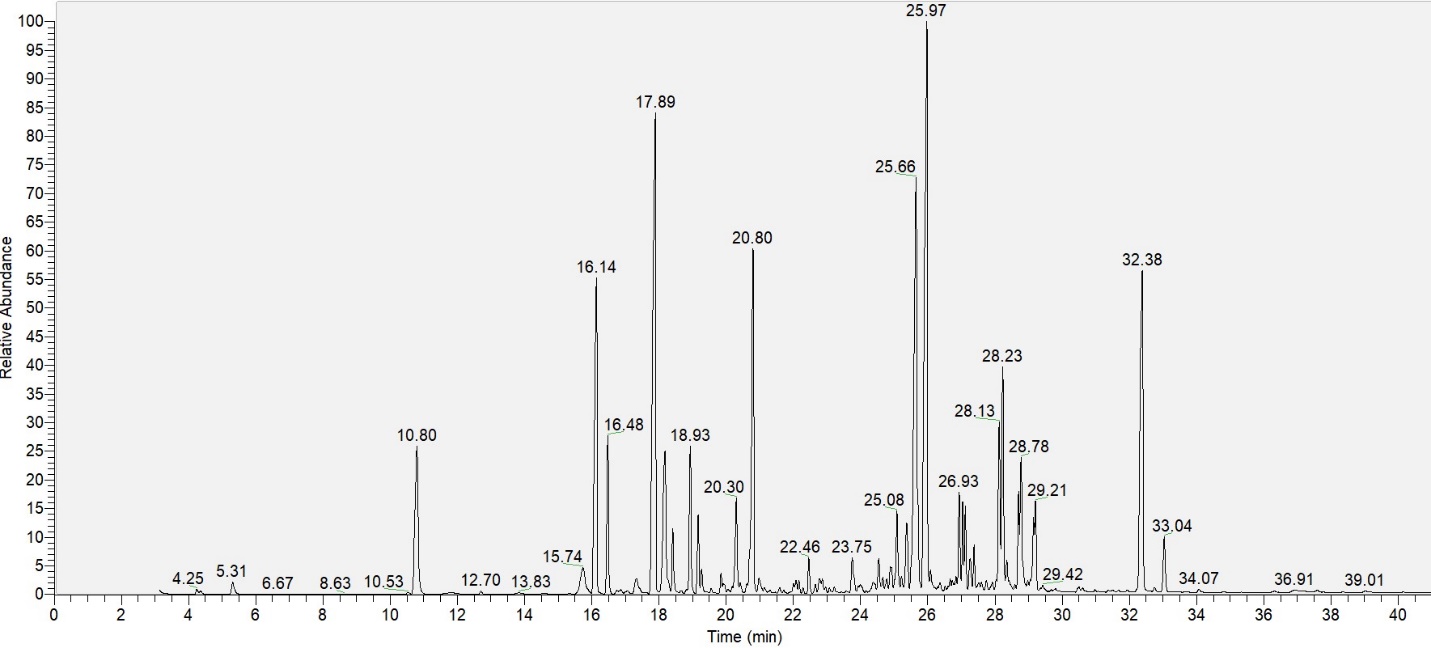


Figure 1S. Chromatogram of the sage essential oil obtained by hydrodistillation at 200 W (sample D 200 W)


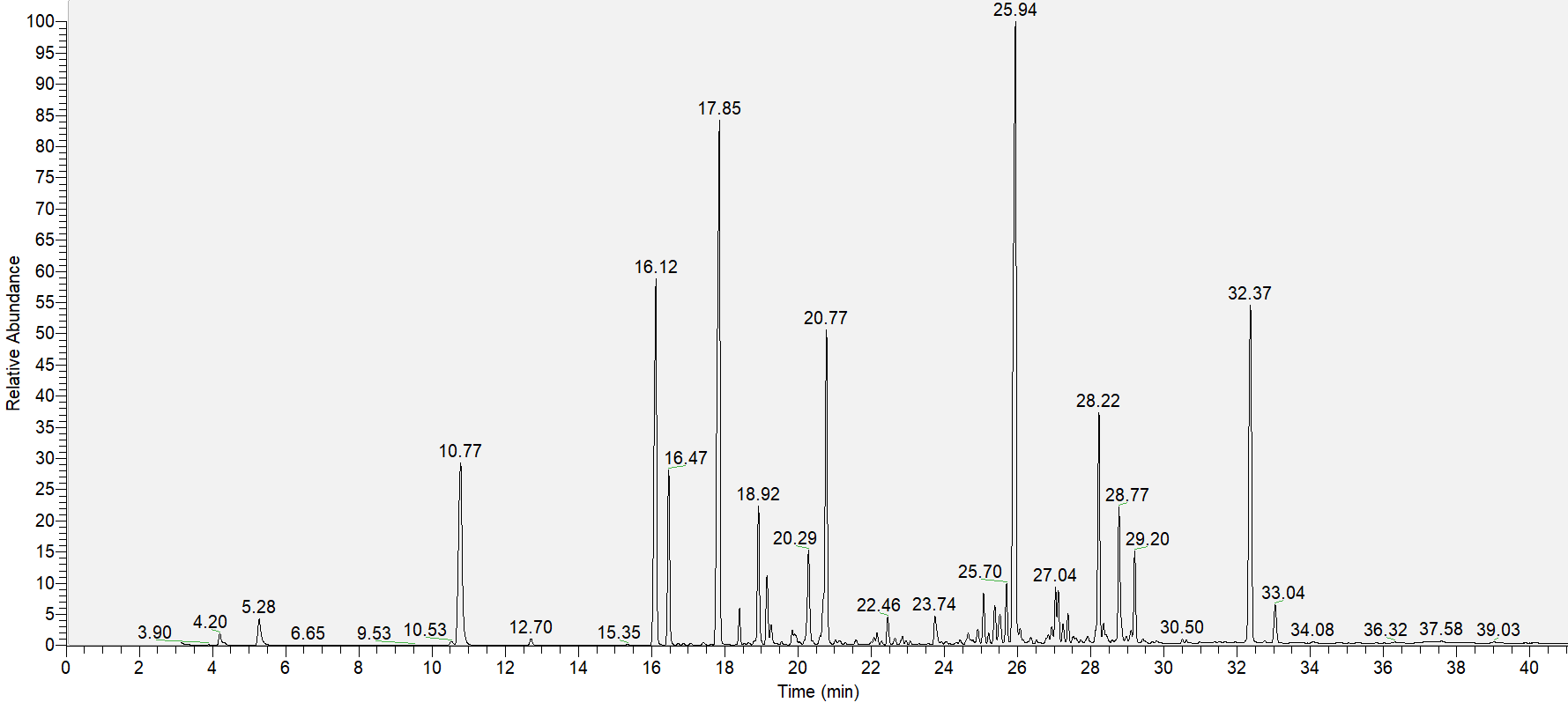


Figure 2S. Chromatogram of the sage essential oil obtained by hydrodistillation at 400 W (sample D 400 W)


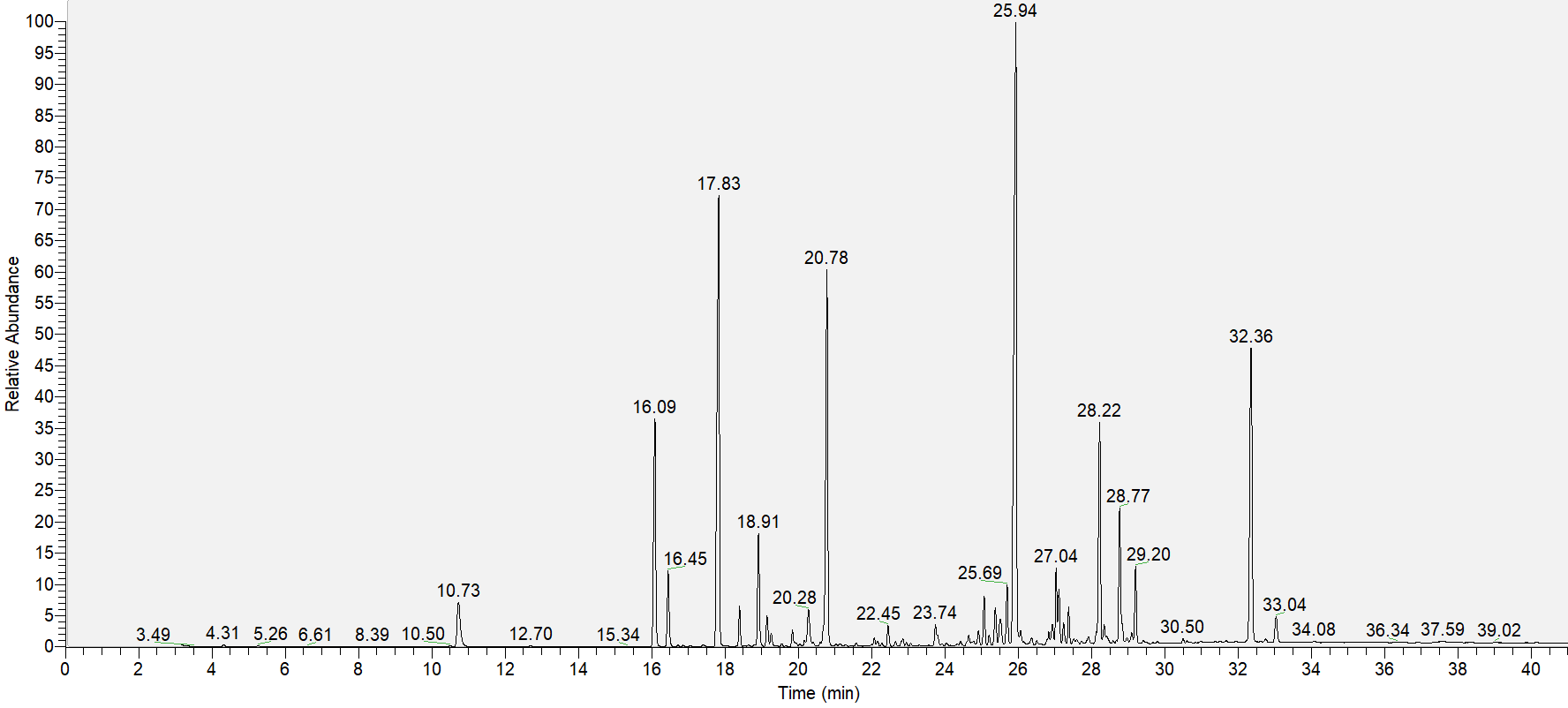


Figure 3S. Chromatogram of the sage essential oil obtained by microwave-assisted hydrodistillation at 200 W (sample MWD 200 W)


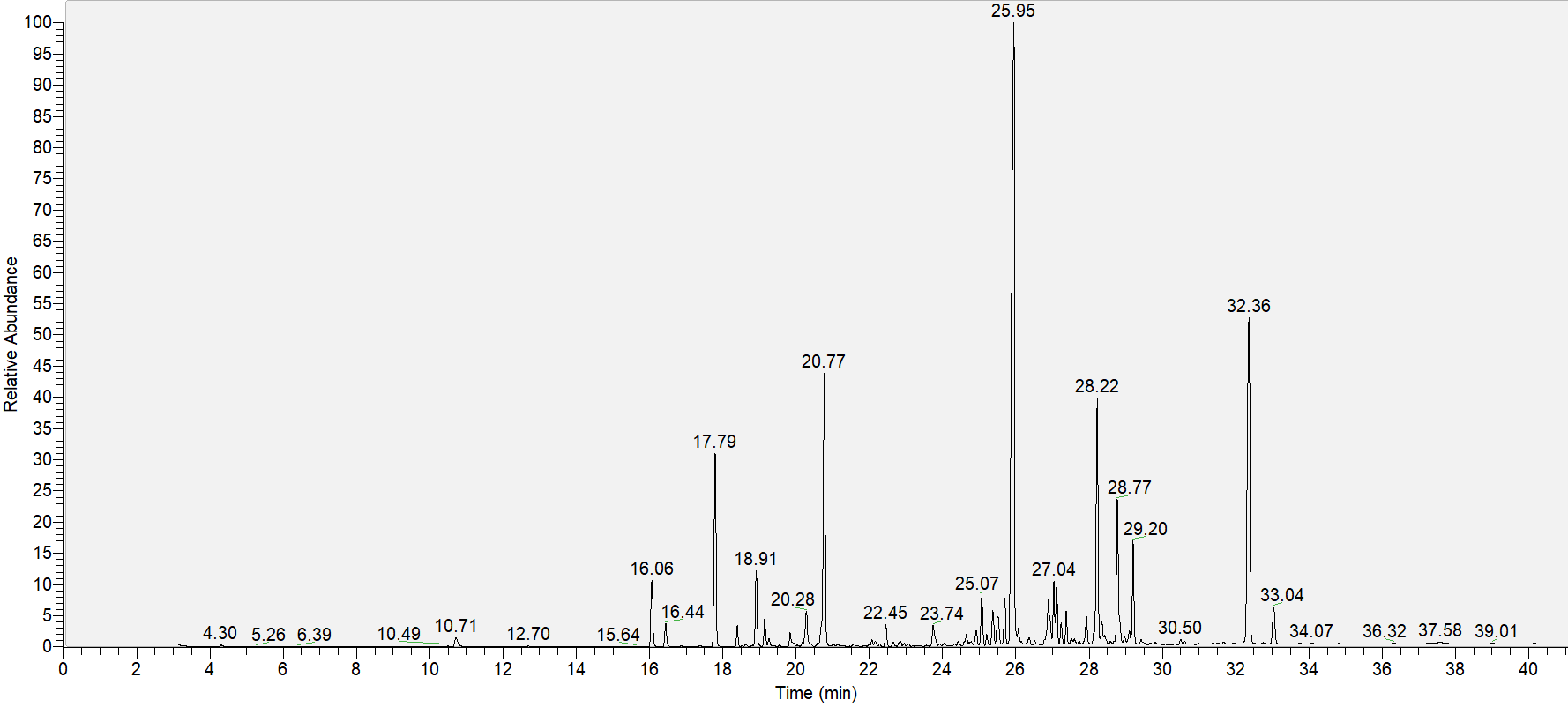


Figure 4S. Chromatogram of the sage essential oil obtained by microwave-assisted hydrodistillation at 400 W (sample MWD 400 W)


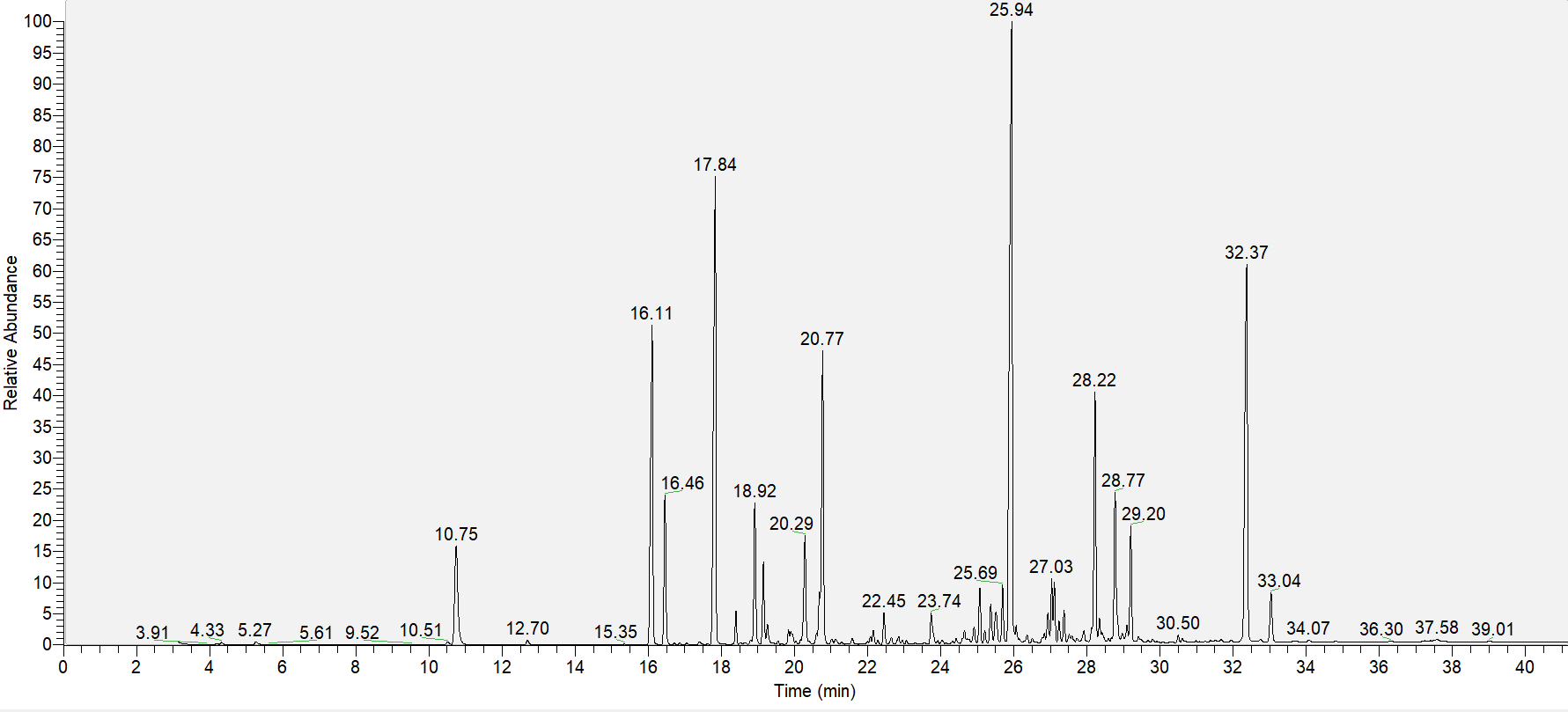


Figure 5S. Chromatogram of the sage essential oil obtained by microwave-assisted hydrodistillation at 600 W (sample MWD 600 W)


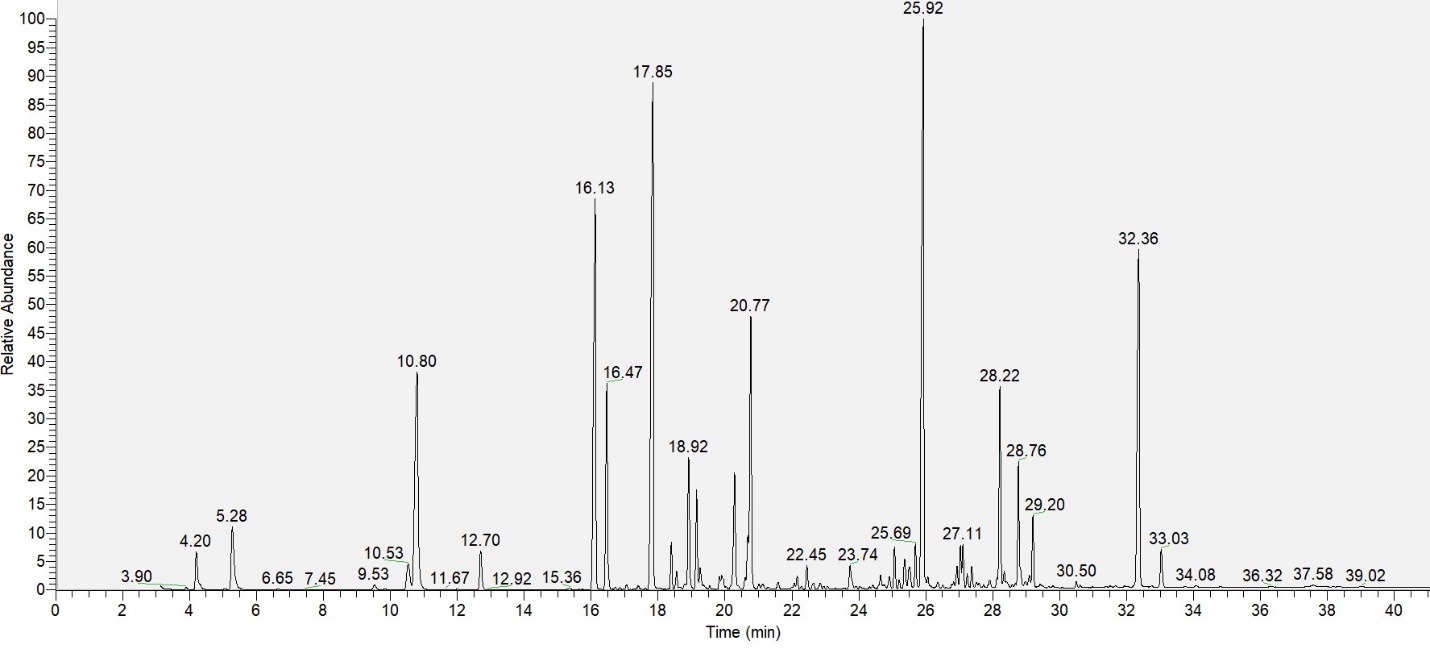


Figure 6S. Chromatogram of the sage essential oil obtained by microwave-assisted hydrodistillation at 800 W (sample MWD 800 W)
